# Gut bacterium *Delftia tsuruhatensis* strain ALG19 isolated from *Agrilus planipennis* larvae degrades cellulose in *Fraxinus velutina*

**DOI:** 10.3389/fmicb.2025.1567054

**Published:** 2025-04-01

**Authors:** Yi-Han Wang, Qi Liu, Thi Minh Dien Vuong, Hua-Ling Wang, Jing-Yi Fu, Xiao-Yu Su, Ying-Jie Wang, Jia-Yu Yang, Jian-Yong Zeng, Hui-Ping Li

**Affiliations:** ^1^Department of Forest Protection, College of Forestry, Hebei Agricultural University, Baoding, China; ^2^Modern Educational Technology Center, Hebei Agricultural University, Baoding, China; ^3^Hebei Urban Forest Health Technology Innovation Center, Baoding, China; ^4^Hebei Key Laboratory for Tree Genetic Resources and Forest Protection, Baoding, China

**Keywords:** *Agrilus planipennis*, gut bacteria, *Delftia tsuruhatensis*, cellulose degradation, *Fraxinus velutina*

## Abstract

**Introduction:**

Strain ALG19, a predominant culturable bacterium isolated from the larval gut of the emerald ash borer (*Agrilus planipennis*) infesting velvet ash (*Fraxinus velutina*), was investigated to determine its taxonomic identity and evaluate its cellulose-degrading potential.

**Methods:**

The taxonomic classification of ALG19 was determined through whole-genome sequencing, average nucleotide identity (ANI) analysis, and phylogenetic reconstruction based on single-copy orthologous genes. Functional annotation of carbohydrate-active genes was performed using the COG, KEGG, and CAZy databases. Cellulolytic activity was assessed using a multi-faceted approach. First, carboxymethyl cellulose hydrolysis assays were conducted to evaluate cellulolytic capability. Additionally, filter paper degradation and the utilization of velvet ash phloem cellulose were examined. For these experiments, the strain was cultured in an inorganic salt medium supplemented with the respective cellulose substrates for 60 days.

**Results:**

Genomic analyses confirmed that ALG19 belongs to *Delftia tsuruhatensis*. The strain harbors 283 COG-annotated genes associated with carbohydrate transport and metabolism, 355 KEGG genes involved in carbohydrate metabolism pathways, and 105 CAZy-annotated carbohydrate-active enzymes. Phenotypic assays revealed a carboxymethyl cellulose hydrolysis zone ratio of 1.74. After a 60-day incubation period, ALG19 completely decomposed filter paper strips into flocs, resulting in a 38.06% reduction in dry weight compared to control samples, which basically retained their original shape. Furthermore, the strain degraded velvet ash phloem cellulose, leaving a residual content of 69.91%. This was 15.60% lower than the control, which exhibited a residual content of 82.83%.

**Discussion:**

These findings demonstrate that *D. tsuruhatensis* ALG19 is capable of degrading cellulose present in the host plant of the emerald ash borer, its associated insect. This study identifies a potential target microorganism for future pest management strategies, which could mitigate the damage caused by the emerald ash borer by impairing its digestive capacity.

## 1 Introduction

The emerald ash borer (*Agrilus planipennis*), a highly destructive pest, has significantly impacted the economy and ecology of regions where ash trees (*Fraxinus* spp.) are found. Originating in Northeast Asia, its distribution spans China, Japan, and Korea, among other Asian countries (Wei et al., 2004). Through transportation, it has spread to many other countries, including the United States, Russia, and Ukraine. It is now recognized as the most damaging and economically costly invasive forest pest in North America and Europe (Nisbet et al., 2015; Bauer et al., 2015). Furthermore, susceptible ash trees imported from North America, such as velvet ash (*F. velutina*), green ash (*F. pennsylvanica*), and white ash (*F. americana*), have suffered severe damage from the emerald ash borer (Sun et al., 2024). For instance, in Beijing, China, the damaged area of ash forests due to the emerald ash borer reached 210 hectares in 2017 (Bureau Beijing Gardening and Greening, 2017). The emerald ash borer larvae primarily feed on the phloem of ash trees, which contains cellulose, a difficult-to-decompose component of cell walls (Cappaert et al., 2005). Therefore, to feed on ash trees, the emerald ash borer must digest the cellulose present in the trees.

Gut bacteria form a complex micro-ecosystem within insects and have a close symbiotic relationship with them (Pan et al., 2020). Studies have shown that the composition of gut bacteria is influenced by the insect’s diet (Li C. et al., 2022; Li J. et al., 2022). For instance, in the European corn borer (*Ostrinia nubilalis*), a diet of corn has been shown to shape the gut bacterial community, enabling the insect to utilize specific bacteria for the digestion of corn lignocellulose (Li C. et al., 2022; Li J. et al., 2022). These bacteria play a crucial role in nutrient digestion, producing various digestive enzymes that help the host insect degrade indigestible food components, such as cellulose (Jing et al., 2020). By studying the cellulose degradation function of gut bacteria in herbivorous insects, like the emerald ash borer, we can gain a deeper understanding of their adaptability to host plants. Furthermore, research on gut bacteria with cellulose degradation capabilities has broader implications beyond insect biology. These bacteria can also be used directly or indirectly for the degradation of biomass resources like straw (Jeong et al., 2024), and have the potential to reduce pollution from industrial wastewater in aquatic ecosystems by degrading lignin produced in the paper-making industry (Saha et al., 2022). Furthermore, there is potential for developing low-toxicity insecticides that target cellulose-degrading bacteria, thereby weakening the digestive ability of insects.

Current research has revealed that many insect gut bacteria have the ability to degrade cellulose. Using traditional methods, two *Bacillus* strains with cellulose-degrading capabilities were isolated from fall armyworm larvae (*Spodoptera frugiperda*) (Zhang et al., 2023). Similarly, 45 strains with cellulose-degrading capabilities were isolated from the intestines of white-striped long-horned beetle larvae (*Batocera horsfieldi*), with two strains from the genera *Ochrobactrum* and *Raoultella* being particularly efficient cellulase producers (Su et al., 2024). In red palm weevil larvae (*Rhynchophorus ferrugineus*), seven species from the genera *Klebsiella*, *Serratia*, *Enterobacter*, and *Citrobacter* were found to degrade cellulose (Muhammad et al., 2017). Additionally, five bacterial species isolated from termite guts were confirmed to have cellulose-degrading abilities, including *Paenibacillus lactis*, *Lysinibacillus macroides*, *Stenotrophomonas maltophilia*, *Lysinibacillus fusiformis*, and *Bacillus cereus* (Ali et al., 2019). To date, 63 bacterial genera with cellulose-degrading functions have been documented (Liu et al., 2024). However, it is believed that many more insect gut bacteria with this capability remain undiscovered and unused. This has driven continued interest in studying gut bacteria with microbial degradation functions, particularly in insects with strong feeding capacities and high destructive potential.

Carboxymethyl cellulose is frequently used to assess the function of cellulose degradation. It is documented that out of 46 studies focused on gut bacteria in insects with cellulose degradation capabilities, 42 studies utilized carboxymethyl cellulose to verify the degradation function of strains. The remaining studies used filter paper cellulose, microcrystalline cellulose, or food digestibility as indicators (Liu et al., 2024). While carboxymethyl cellulose offers simplicity and feasibility in cellulose degradation tests, it also has limitations. Specifically, although both carboxymethyl cellulose (C_8_H_16_O_8_) and plant cellulose [(C_6_H_10_O_5_)_n_] are polysaccharides, they differ in chemical structure and physical properties (Sawant et al., 2023). Carboxymethyl cellulose is a water-soluble, chemically modified cellulose ether that microorganisms can easily degrade. In contrast, plant cellulose decomposition is influenced by crystallinity and requires specific cellulases (Fu, 2019). Using plant cellulose in research provides a more accurate reflection of gut bacterial ability to digest host plant nutrients in insects. This approach will provide a stronger scientific basis for developing control strategies that target gut bacteria capable of cellulose degradation.

Strain ALG19 is a dominant dominant bacterium among the culturable gut microbiota of emerald ash borer larvae. In this study, taxonomic identification and carbohydrate hydrolysis capability analysis of strain ALG19 were conducted using genome sequencing. To further evaluate its cellulose degradation capabilities, experiments were conducted using carboxymethyl cellulose, filter paper cellulose, and phloem cellulose extracted from velvet ash, the host plant of the emerald ash borer. The ultimate goal of this research was to elucidate the ability of gut bacteria to assist host insects in digesting nutrients, providing a foundation for pest management strategies targeting insect gut bacteria.

## 2 Methods

### 2.1 Genome sequencing of gut bacteria ALG19

The test strain ALG19 was isolated from the guts of the emerald ash borer larvae collected from the infected velvet ash forest in the “Millennium Show Forest” in Xiong’an New Area, China. It was inoculated into the Luria-Bertani liquid medium and cultured with constant temperature shaker at 30°C and 180 r/min until the suspension became turbid. The obtained suspension was used for genomic DNA extraction using the PureLink^®^ Genomic DNA Kit (Invitrogen, CA, United States). Whole-genome sequencing was performed using the Illumina HiSeq novaseq6000 sequencing system (Illumina, CA, United States) and PacBio technology. The sequencing depth of the second-generation data is 158×, and the sequencing depth of the third-generation data is 79×. The genome sequencing work was undertaken by Shanghai Majorbio Bio-Pharm Technology Co., Ltd. (Shanghai, China).

### 2.2 Taxonomic analysis and genomic annotation of gut bacteria ALG19

Genomic information of seven strains of *Delftia tsuruhatensis* were compared with strain ALG19. Those seven strains were isolated from different sources, and their accession in Genbank and source were list in Table 1. Benchmarking Universal Single-Copy Orthologs (BUSCO) was used to evaluate the completeness of bacterial genome (Manni et al., 2021). The final completion map of the sequencing results was drawn by the Prodigal software (Hyatt et al., 2010). The average nucleotide identity (ANI) of the genome was calculated to measure the nucleotide-level genomic similarity among analyzed strains (Kastner et al., 2024). A species tree was generated using OrthoFinder^1^ with the Species Tree of All Genes (STAG) algorithm (Emms and Kelly, 2019), and visualized by the FigTree software^2^ (Boore, 2006). Meanwhile, the genome of strain ALG19 was subjected to COG functional annotation, KEGG metabolic pathway annotation and CAZy gene annotation to predict the potential of strain ALG19 to degrade cellulose. All genome analyses were completed on the Majorbio Cloud Platform (Ren et al., 2022).

**Table 1:** Genome summary of *Deltftia tsuruhatensis* from different sources and strain ALG19.

Strain	Accession	Size (Mb)	GC percent (%)	Protein-coding	ANI value (%)	BUSCO value (%)	Source
CM13	GCF_001753225.1	7.2	66.5	6,554	98.60	99.5	Mice
TR1180	GCF_009362995.1	6.7	66.5	5,935	98.52	98.8	Human
ULwDis	GCF_029215875.1	6.9	66.5	6,302	99.45	98.5	Seawater
Ery-6A	GCF_029590315.1	6.7	66.5	5,901	98.68	99.7	Soil
GD03927	GCF_029814915.1	6.8	66.5	6,094	98.64	99.8	Hospital sink
GSK TC1	GCF_036828685.1	6.8	66.5	5,986	98.67	98.8	Mosquito
BB1455	GCF_903815225.1	6.1	67	5,419	90.14	99.3	Wastewater

### 2.3 Carboxymethyl cellulose degradation capacity evaluation of gut bacteria ALG19

A single colony of the ALG19 strain was inoculated into the Luria-Bertani liquid medium and then cultured at 30°C and 180 r/min for 24 h to obtain the bacterial suspension, which was subsequently used for the determination of carboxymethyl cellulose degradation capacity as previously reported method (Li et al., 2021). Take 18 mL of the carboxymethyl cellulose Congo red medium and pour it into a Petri dish with diameter of 9 cm. After the medium has solidified, use a puncher with diameter of 8 mm to create two symmetrical holes. Subsequently, add 70 μL of sterile water (used as the control) and the suspension of strain ALG19, respectively. The Petri dish was sealed with parafilm and then incubated at 30°C for 3 days. Three biological replicates were performed to eliminate errors. The ratio of the diameter of the degradation circle (D) to the diameter of the punched hole (d) was calculated to evaluate the degradation capacity of the ALG19 strain on carboxymethyl cellulose.

### 2.4 Filter paper cellulose degradation capacity evaluation of gut bacteria ALG19

An inorganic salt liquid medium was prepared according to the following formulation: 1 g of (NH_4_)_2_SO_4_, 3 g of KH_2_PO_4_, 7 g of K_2_HPO_4_, 0.1 g of MgSO_4_·7H_2_O, and 1,000 mL of H_2_O. A single colony of strain ALG19 was inoculated into 25 mL of inorganic salt liquid medium, and then cultured at 30°C and 180 r/min for 12 h. The resulting bacterial suspension was used to assess the ability of strain ALG19 to degrade both filter paper cellulose and phloem cellulose extracted from velvet ash, the host plant of the emerald ash borer. Subsequently, a conical flask with 100 mL of inorganic salt medium was prepared and mixed with 2 mL of bacterial suspension and 3 sterile filter paper strips (1 cm × 4 cm, 0.04 g each). The mixture was incubated at 30°C and 180 r/min for 60 days. A control was prepared using bacterial suspension sterilized at 121°C under high pressure steam for 30 min. The experiment was repeated three times. The filter paper’s disintegration was observed, the residue was filtered, dried at 50°C, weighed, and the degradation rate was calculated to study the degradation capacity of the ALG19 strain on filter paper cellulose.

### 2.5 Velvet ash cellulose degradation capacity evaluation of gut bacteria ALG19

Cellulose extracted from the phloem of velvet ash was also used to test the degradation capacity of strain ALG19. The phloem was collected from healthy plants using a peeling knife, washed with distilled water to remove impurities, and dried at 60°C until it reached a constant weight. The dried phloem was pulverized into powder and passed through a 100-mesh sieve for cellulose extraction. To extract the cellulose, an optimized HNO_3_-NaOH method was employed (Wen et al., 2017). The procedure involved mixing the phloem powder with a 3% HNO_3_ solution at a liquid-to-solid ratio of 35 mL/g and heating it at 90.7°C for 2.9 h. The mixture was then filtered and washed until it reached a neutral pH. An equal volume of 3% NaOH solution was added to the acid-treated phloem, and the mixture was heated at 90.7°C for another 1.5 h. Once cooled to about 75°C, H_2_O_2_ was introduced to reach a concentration of 5%, and the heating continued for an additional 0.5 h. The extracted cellulose was then filtered, washed with distilled water until neutral pH, dried at 60°C, and sterilized at 121°C for 30 min.

For the degradation assay, 0.5 g of the sterilized ash cellulose was mixed with 25 mL of inorganic salt medium and 2 mL of the bacterial suspension of strain ALG19. Subsequently, the resulting mixture was incubated at 30°C and 180 r/min for 60 days. After incubation, the mixture was centrifuged at 8,000 r/min for 10 min to obtain the precipitate. The precipitate was then dried at 70°C until it reached a constant weight. The cellulose content in the precipitate was measured using a cellulose content kit (Grace Bio-Tech, Suzhou, China), and the degradation rate of the ash cellulose was calculated to evaluate the degradation capacity of strain ALG19. A control was prepared using an equal volume of bacterial suspension inactivated at 121°C for 30 min. The experiment was repeat three times to reduce error.

### 2.6 Statistical analysis

Variance homogeneity and normal distribution were assessed to determine the suitability of parametric or non-parametric tests. Based on the results, the relationship between the hydrolysis circle of strain ALG19 and the diameter of the punched hole was conducted using Kruskal–Wallis tests. Additionally, the dry weight of the centrifugal precipitate from filter paper strips and the cellulose content in the extracted phloem cellulose from velvet ash were analyzed using Student’s *t*-tests. All statistical analyses were performed using IBM SPSS Statistics 21, and significance was set at *p* < 0.05.

## 3 Results

### 3.1 Taxonomic status of gut bacteria ALG19

**Figure 1:**
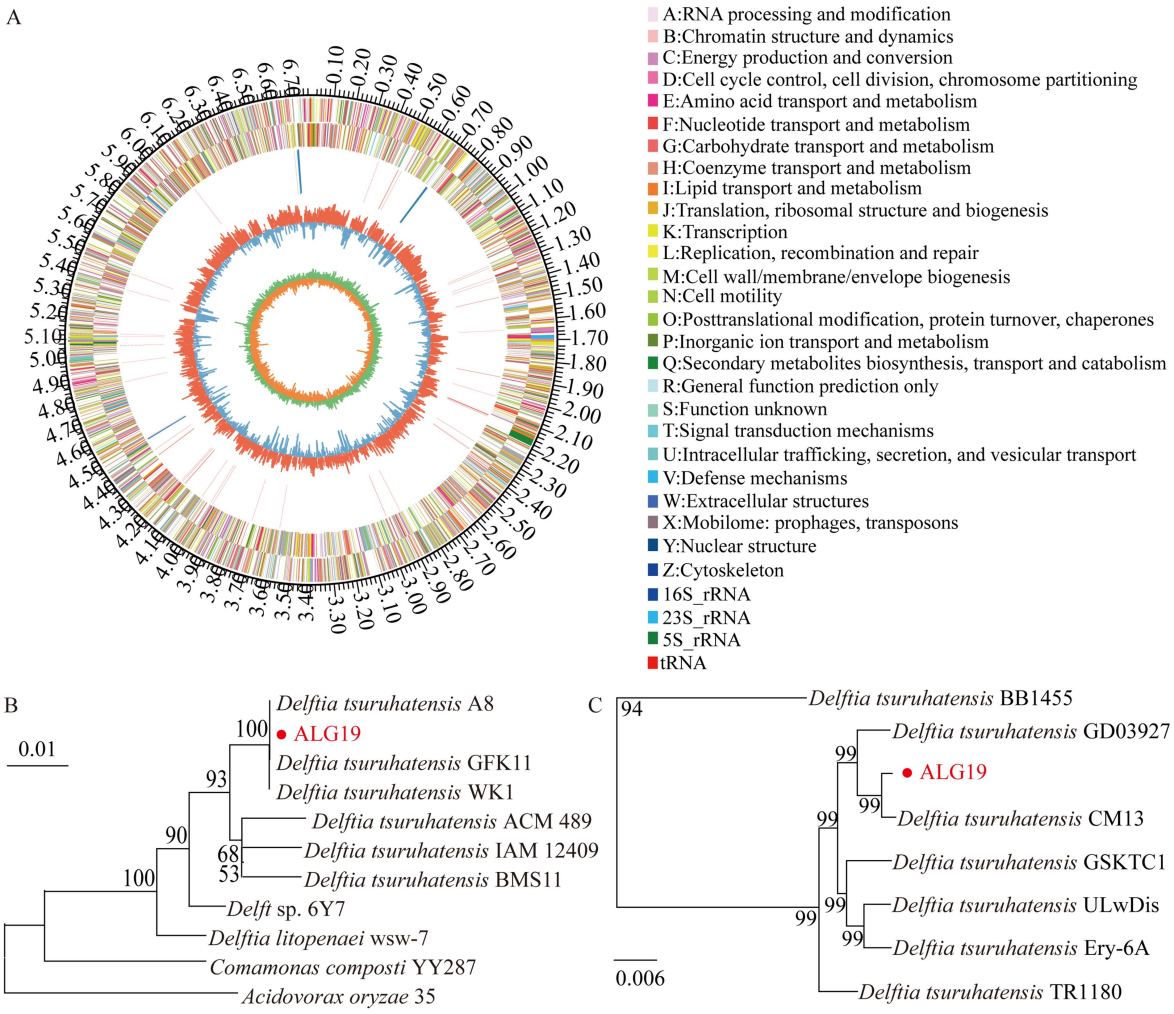
Genomic analysis of strain ALG19. **(A)** Circos plot illustrating the genomic features of strain ALG19. **(B)** Phylogenetic tree showing the genetic relationship between strain ALG19 and other related strains, as inferred from 16S rRNA sequence data. **(C)** Phylogenetic analysis based on single-copy orthologous genes, including strain ALG19 and seven *D. tsuruhatensis* strains.

The genome of strain ALG19 is 6,796,678 base pairs long. It contains 6,146 coding genes, which total 6,092,280 base pairs in length, with an average gene length of 991 bp. The genome has a GC content of 67.43%. Figure 1A illustrates the genomic features of ALG19 using a Circos plot. To determine its phylogenetic position, the 16S rRNA sequence of ALG19 was used to build a phylogenetic tree (Figure 1B), preliminarily identifying it as *D. tsuruhatensis*. To further confirm its classification, seven *D. tsuruhatensis* strains isolated from diverse environments—including mice, humans, seawater, soil, hospital sink, the mosquito *Anopheles stephensi*, and wastewater—were chosen for comparative genomic studies. These strains had similar genome sizes, a comparable number of protein-coding genes, and GC contents ranging from 66.5 to 68%. Additionally, their BUSCO assembly quality scores were all above 98%, making them suitable for the analysis. The analysis revealed that except BB1455 from wastewater, which had an ANI value of only 90.14%, other strains had ANI values above 98.50% when compared to ALG19. A phylogenetic tree based on single-copy orthologous genes (Figure 1C) further confirmed that ALG19 grouped with the other six *D. tsuruhatensis* strains, excluding BB1455. This evidence strongly supports the classification of strain ALG19 as part of the *D. tsuruhatensis* species.

### 3.2 Carbohydrate degradation function annotation of gut bacteria strain ALG19

**Figure 2:**
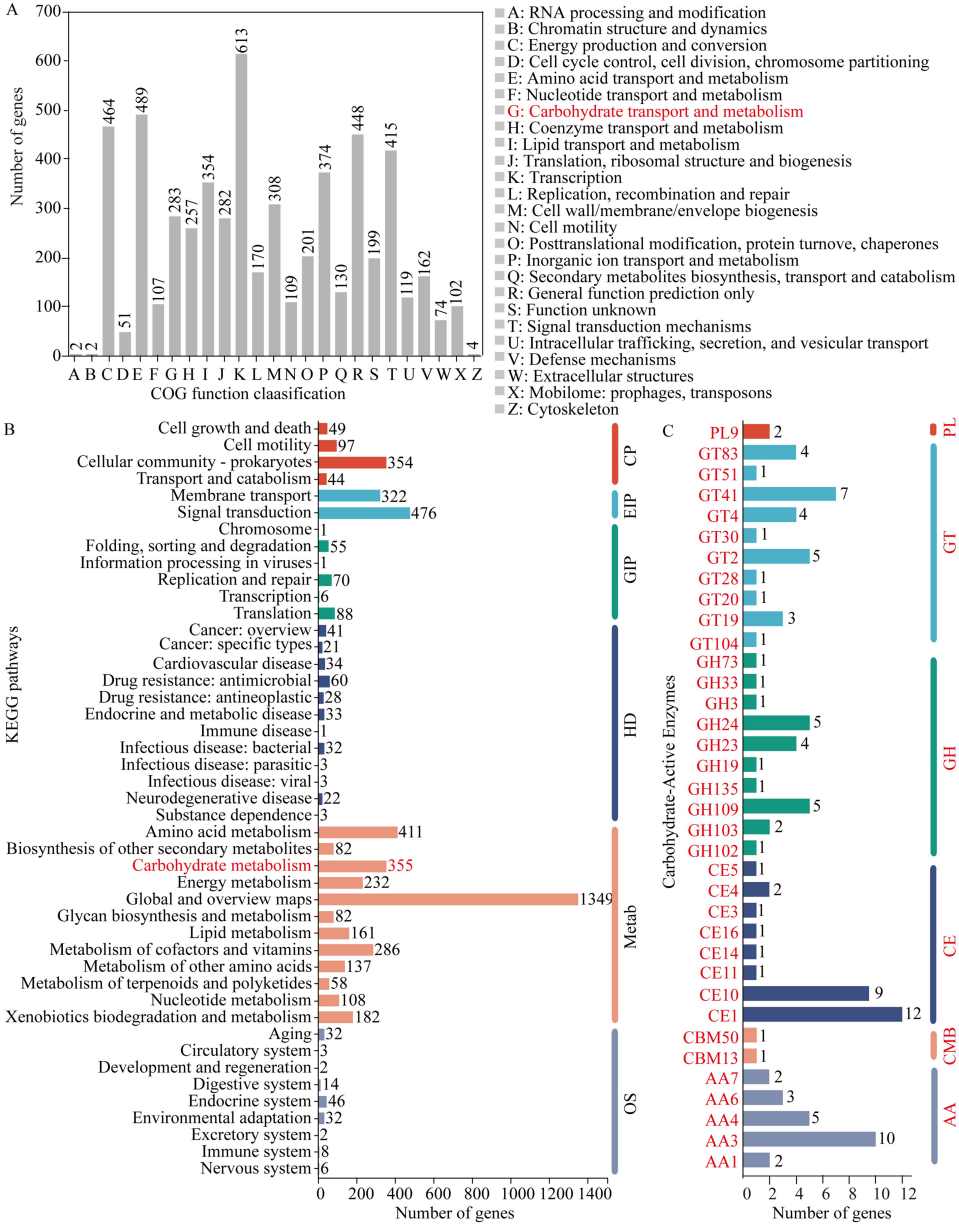
Gene annotation results for strain ALG19. **(A)** Annotation of COG function clusters. **(B)** Annotation of KEGG pathways. **(C)** Annotation within the CAZy database. **(B)** The clusters include: CP for cellular processes, EIP for environmental info processing, GIP for genetic info processing, HD for human diseases, Metab for metabolism, and OS for organismal systems. **(C)** Displays the count of genes annotated within gene families including glycoside hydrolase (GH), glycosyltransferase (GT), carbohydrate esterase (CE), carbohydrate-binding module (CBM), polysaccharide lyase (PL), and auxiliary activity (AA).

A total of 5,084 genes were categorized into 25 functional clusters in the COG database. Notably, no genes were assigned to the nuclear structure cluster (cluster Y). Among the annotated genes, 283 were associated with the carbohydrate transport and metabolism cluster (cluster G). In terms of gene count, cluster G ranked ninth, with eight other clusters having a higher number of annotated genes, ranging from 308 to 613 genes (Figure 2A). In the KEGG database, 4,630 genes were annotated. Of these, 3,443 genes related to metabolism, comprising 74.36% of the annotated genes and dominating the biological processes category. Within the metabolism-related genes, 355 were associated with the carbohydrate metabolism pathway. In terms of gene abundance, 476 genes were associated with carbohydrate metabolism, while 1,349 genes were linked to the global and overview maps pathway (Figure 2B).

Annotation within the CAZy database revealed 105 carbohydrate-active enzyme genes in *D. tsuruhatensis* ALG19, including 22 glycoside hydrolase (GH) genes, 28 glycosyltransferase (GT) genes, 29 carbohydrate esterase (CE) genes, 2 carbohydrate-binding module (CBM) genes, 2 polysaccharide lyase (PL) genes, and 22 auxiliary activity (AA) genes. Furthermore, the GH genes were classified into 10 families, with the GH109 family having five genes, the GH24 family having five genes, and the GH23 family having four genes. The GT genes were distributed across 10 families, with the GT41 family being the most prevalent with seven genes. The CE genes were categorized into eight families, with the CE1 family having the largest number at 12 genes. The two CBM genes were from the CBM13 and CBM50 families, respectively, and both PL genes belonged to the PL9 family. The AA genes were divided into five families, with the AA3 family having the highest count of 10 genes (Figure 2C). The collective annotation results from the COG, KEGG, and CAZy databases strongly support the capability of *D. tsuruhatensis* ALG19 to degrade cellulose.

### 3.3 Cellulose degradation efficiency of strain ALG19 on three cellulose variants

**Figure 3:**
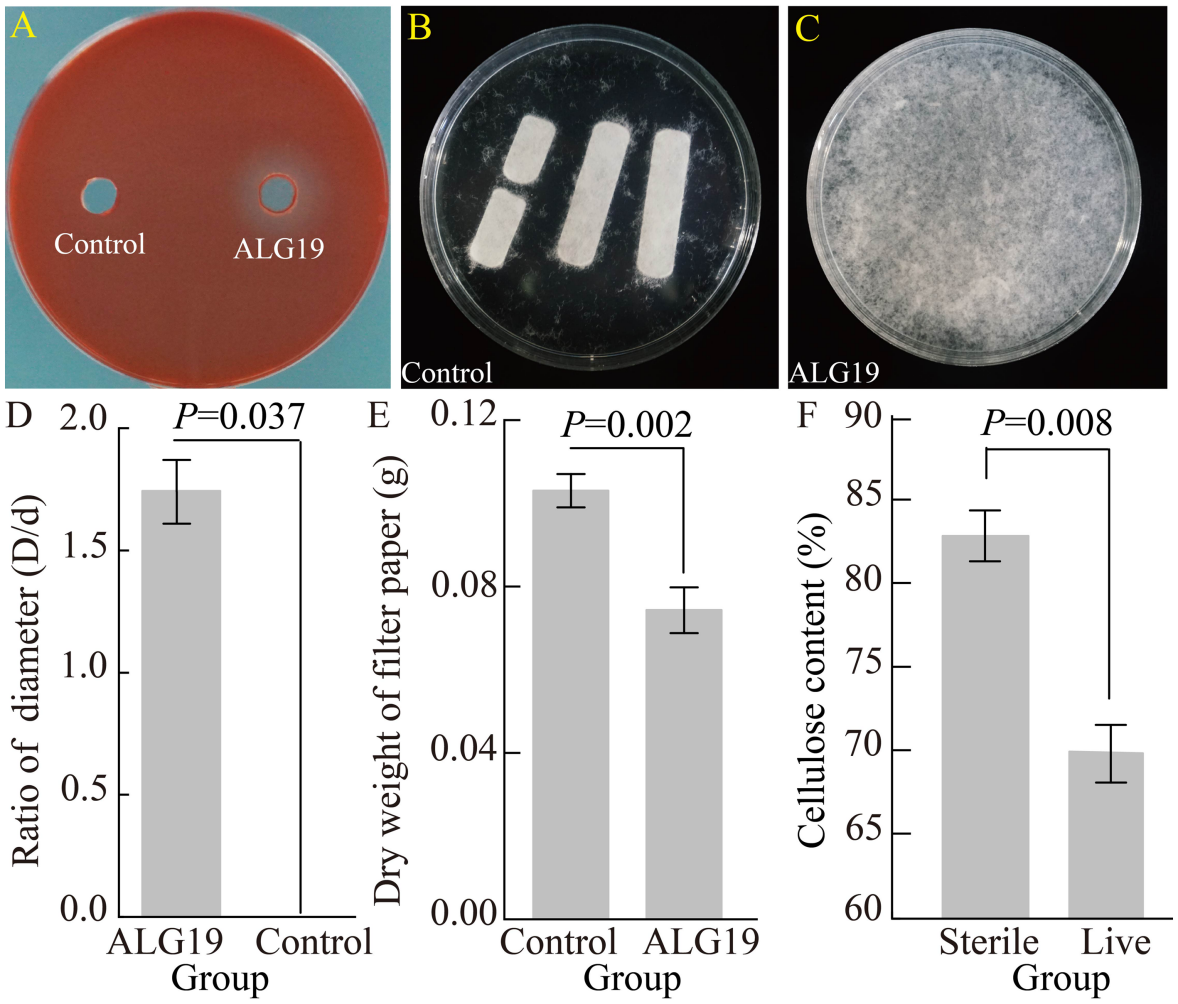
Cellulose degradation efficiency of strain ALG19 on three cellulose variants. **(A,E)** Demonstrate its ability to degrade carboxymethyl cellulose. **(C,D)** Its capacity to degrade filter paper cellulose, with **(B)** serving as a control for the filter paper. The final panel **(F)** illustrates ALG19’s ability to degrade the cellulose found in the phloem of the host plant, velvet ash. The data are expressed as mean values, with the error bars in the figures indicating the standard error.

The Congo red assay showed that strain ALG19 produced a conspicuous hydrolysis circle, but no such prominent circle was observed in the control group. The diameter ratio of the hydrolysis circle of strain ALG19 to the diameter of the punched hole was 1.74, which was markedly higher than that in the control group (df = 1, χ^2^ = 4.355, *p* = 0.037). This finding suggests that *D. tsuruhatensis* strain ALG19 possesses the capacity to degrade carboxymethyl cellulose (Figures 3A,D).

The filter paper cellulose degradation experiment indicated that following a 60-day incubation in the inorganic salt medium, the filter paper strips in the control group presented rough edges, disintegration at corners, and central splits, while still largely maintained their initial shape. In contrast, inoculation with live strain ALG19 result in significant pronounced disintegration, with the filter paper strips completely broken down into flocculent particles. The dry weight of the centrifugal recovery product in the treatment group was 0.074 g, significantly lower than the 0.103 g in the control group (df = 4, *t* = 7.294, *p* = 0.002). The disintegration rate attained 38.06%, which was 2.69 times that of the control group. Consequently, it can be inferred that *D. tsuruhatensis* ALG19 exhibits a capacity to degrade filter paper cellulose (Figures 3B,C,E).

Subsequent tests of degradative capability revealed that after 60 days of co-cultivation in an inorganic salt medium, the cellulose content in the extracted phloem cellulose from velvet ash was reduced to 69.91% when inoculated with the live strain ALG19. This was significantly lower than the 82.83% observed in the control group (Figure 3F, df = 16, *t* = 3.028, *p* = 0.008). The calculated degradation rate was 15.60%. These results indicate that the gut bacterium *D. tsuruhatensis* ALG19, isolated from emerald ash borer larvae, has the ability to degrade the cellulose in the phloem of the host plant, velvet ash.

## 4 Discussion

Investigations into the gut microbiota of the emerald ash borer have revealed that the bacterial constituents predominantly consist of genera within the class Gammaproteobacteria in adult emerald ash borers, such as *Pantoea*, *Escherichia*, *Shigella*, *Pseudomonas*, and *Chryseobacterium*. Notably, *Pantoea* has been identified as the dominant bacterial genus in the gut of the emerald ash borer (Mogouong et al., 2020). Furthermore, studies focusing on the emerald ash borer larvae have revealed that five bacterial phyla and 39 bacterial families have been detected, including Pseudomonadaceae, Erwiniaceae, and Enterobacteriaceae (Vecherskii et al., 2022). *Pseudomonas* was the dominant genus during the larval phase (Vasanthakumar et al., 2008). This study focus on the culturable bacterial strain ALG19, which is present in the gut of the emerald ash borer larvae. It exhibits the highest isolation rate in our previous culturable gut bacteria isolation experiments, prompting further investigation. The strain was identified as *D. tsuruhatensis* based on genome sequencing.

The genus *Delftia* was established as a novel bacterial genus in 1999 by Australian scholar Wen and colleagues (Wen et al., 1999). It was established through the analysis of 16S rRNA gene sequences, resulting in the segregation of *Comamonas acidovorans* from the genus *Comamonas*. From a phylogenetic perspective, the genus *Delftia* is taxonomically classified within the family Comamonadaceae, order Burkholderiales, *β*-subclass of the Proteobacteria. It is worth noting that *D. tsuruhatensis* is not a newly discovered bacterial species in insect guts. In fact, it has been previously isolated from the gut of *Anopheles mosquitoes* (Pierre et al., 2023). Furthermore, existing research has demonstrated that *D. tsuruhatensis* XSP-1, isolated from the sludge of a pesticide factory, exhibits the capacity to degrade pesticides such as methyl parathion, chlorpyrifos, and fenitrothion (Han et al., 2005). Additionally, *D. tsuruhatensis* AD4, sourced from activated sludge, has been shown to possess a high proficiency in degrading the toxic substance aniline (Yuan et al., 2023). These findings suggest that *D. tsuruhatensis* may assist insects in adapting to the stress induced by toxic organic compounds, including pesticides. It is also conceivable that this bacterium could be implicated in the adaptation of the emerald ash borer to the defensive substances produced by host plants.

Nutrient digestion is a crucial function of insect gut bacteria (Holtof et al., 2019). However, our literature review revealed no studies on the cellulose degradation capacity of *D. tsuruhatensis*. Nevertheless, the emerald ash borer’s ability to digest indigestible substances such as cellulose is essential for its feeding on host plants. This prompted us to investigate the cellulose degradation capacity of *D. tsuruhatensis* ALG19. Building upon previous research, the current study has not only confirmed the ability of strain ALG19 to degrade carboxymethyl cellulose and filter paper cellulose but has also demonstrated its capacity to degrade the phloem cellulose of velvet ash, a preferred host plant of the emerald ash borer. The degradation of velvet ash phloem cellulose suggests that *D. tsuruhatensis* ALG19 may enhance the emerald ash borer’s digestive capacity and potentially facilitate its adaptation to the host plant. This discovery provides a fundamental basis for a pest management strategy that aims to impair or eliminate the insects’ digestive capabilities by targeting gut bacteria with cellulose degradation capacity (Siddiqui and Khan, 2023). Furthermore, *D. tsuruhatensis* ALG19 may have direct or indirect applications in the degradation of lignin produced by the paper-making industry, thereby mitigating pollution of aquatic ecosystems from industrial wastewater (Li et al., 2023), or in the degradation of biomass resources such as straw (Chen et al., 2024).

One of the key features of this study is the verification of the cellulose degradation capacity using phloem cellulose extracted from the host plant of the emerald ash borer. This approach differs from previous studies that predominantly relied on carboxymethyl cellulose and filter paper cellulose as substrates (Liu et al., 2024). It is worth noting that different types of celluloses exhibit variations in both chemical structure and physical properties (Sawant et al., 2023). By using the phloem cellulose of the host plant as the exclusive carbon source for validating cellulose degradation, this study provides more reliable evidence regarding the nutrient digestion capacity of insect gut bacteria. However, despite our efforts to screen and optimize the reported cellulose extraction method (results not presented), the purity of the extracted phloem cellulose of velvet ash only reached 84.78%, which is still far below our ideal target of >90% purity. Additionally, in the degradation assays of filter paper cellulose and velvet ash cellulose conducted in this study, the degradation rates were recorded as 38.06 and 15.60% respectively, following a 60 d incubation period. This relatively low degradation rate can be attributed to the choice of medium. To avoid the confounding effects of a nutrient-rich environment, an oligotrophic inorganic salt medium was chosen, with filter paper cellulose or velvet ash cellulose serving as the sole carbon source. This significantly limited the growth of *D. tsuruhatensis* ALG19 and the degradation rates. The 60-day incubation period, while necessary to observe measurable degradation under oligotrophic conditions, may have further constrained the degradation efficiency due to the prolonged nutrient limitation and potential accumulation of metabolic by-products that could inhibit bacterial activity. Therefore, future studies could explore shorter incubation periods with optimized inorganic salt media that better support the growth of strain ALG19, to more accurately assess its cellulose degradation potential.

Furthermore, this study focuses solely on the cellulose degradation function of *D. tsuruhatensis* ALG19, thus providing an incomplete picture of its ecological role. As previously mentioned, the capacity of other *D. tsuruhatensis* strains to degrade toxic substances may assist the emerald ash borer in adapting to the defensive compounds of the host plant. However, further research is needed to verify these inferences and identify the specific defensive substances of the host plant that *D. tsuruhatensis* can degrade. Previous research has also shown that *D. tsuruhatensis* NF83-1 exhibits pronounced inhibitory effects against pathogenic fungi such as *Rhizoctonia solani*, *Alternaria brassicae*, and *Sclerotinia sclerotiorum*, as well as pathogenic bacteria including *Xanthomonas oryzae* pv. *oryzicola*, *Erwinia amylovora*, and *Ralstonia solanacearum* (Chen et al., 2024). This suggests that *D. tsuruhatensis* has potential as a biological agent for plant diseases. Therefore, the ecological role and functional attributes of *D. tsuruhatensis* ALG19 in nature warrant further in-depth research and exploration.

Given the multifaceted capabilities of *D. tsuruhatensis*, its potential applications extend beyond plant disease control to pest management. In the field of pest management, future research could focus on identifying the key enzymes in the ALG19 strain responsible for degrading ash cellulose and developing specific cellulase inhibitors targeting ALG19’s enzymatic machinery. Such efforts would provide a foundation for designing precision inhibitors to block symbiotic cellulose digestion in the ash borer gut ecosystem, thereby disrupting larval nutrient acquisition. This approach mirrors the developmental trajectory of Ledprona, the first sprayable RNA interference (RNAi) biopesticide. Before Ledprona’s introduction, RNAi lacked practical pest control applications, but its success demonstrated how foundational research on nucleic acid delivery mechanisms enabled market-ready solutions. Although still in the research phase, this approach aligns with emerging trends in microbiome-mediated pest management, highlighting its potential for future applications.

## 5 Conclusion

The bacterial strain ALG19, isolated from the gut of emerald ash borer larvae, was identified as *D. tsuruhatensis*. Genomic annotations in the COG, KEGG, and CAZy databases confirm the capability of *D. tsuruhatensis* ALG19 to degrade cellulose. Further assays confirmed that strain ALG19 possesses the ability to degrade carboxymethyl cellulose, filter paper cellulose, and phloem cellulose from velvet ash. Future studies should develop specific cellulase inhibitors targeting ALG19’s enzymatic machinery, which could disrupt larval nutrient acquisition by blocking symbiotic cellulose digestion in the gut ecosystem.

## Data Availability

The raw sequence data were deposited in the National Center for Biotechnology Information (NCBI) Sequence-Read Archive (SRA) database under accession number PRJNA1205903.

## Footnotes

^1^ https://github.com/davidemms/OrthoFinder

^2^ https://github.com/rambaut/figtree/
